# Innovation and Application of the Type III Secretion System Inhibitors in Plant Pathogenic Bacteria

**DOI:** 10.3390/microorganisms8121956

**Published:** 2020-12-09

**Authors:** Xiaochen Yuan, Manda Yu, Ching-Hong Yang

**Affiliations:** 1 Department of Plant, Soil, and Microbial Sciences, Michigan State University, East Lansing, MI 48824, USA; yuanxia9@msu.edu; 2 Department of Biological Sciences, University of Wisconsin-Milwaukee, Milwaukee, WI 53211, USA

**Keywords:** T3SS, plant bacterial diseases, *Dickeyadadantii*, fire blight, T3SS inhibitors, c-di-GMP

## Abstract

Many Gram-negative pathogenic bacteria rely on a functional type III secretion system (T3SS), which injects multiple effector proteins into eukaryotic host cells, for their pathogenicity. Genetic studies conducted in different host-microbe pathosystems often revealed a sophisticated regulatory mechanism of their T3SSs, suggesting that the expression of T3SS is tightly controlled and constantly monitored by bacteria in response to the ever-changing host environment. Therefore, it is critical to understand the regulation of T3SS in pathogenic bacteria for successful disease management. This review focuses on a model plant pathogen, *Dickeya* *dadantii*, and summarizes the current knowledge of its T3SS regulation. We highlight the roles of several T3SS regulators that were recently discovered, including the transcriptional regulators: FlhDC, RpoS, and SlyA; the post-transcriptional regulators: PNPase, Hfq with its dependent sRNA ArcZ, and the RsmA/B system; and the bacterial second messenger cyclic-di-GMP (c-di-GMP). Homologs of these regulatory components have also been characterized in almost all major bacterial plant pathogens like *Erwinia* *amylovora*, *Pseudomonas* *syringae*, *Pectobacterium* spp., *Xanthomonas* spp., and *Ralstonia* spp. The second half of this review shifts focus to an in-depth discussion of the innovation and development of T3SS inhibitors, small molecules that inhibit T3SSs, in the field of plant pathology. This includes T3SS inhibitors that are derived from plant phenolic compounds, plant coumarins, and salicylidene acylhydrazides. We also discuss their modes of action in bacteria and application for controlling plant diseases.

## 1. Introduction

Type III secretion systems (T3SSs) are well-studied protein secretion/translocation systems found in almost all Gram-negative bacterial pathogens of plants and animals [1,2]. Structurally, the T3SSs are highly conserved among bacteria. They are syringe-like nanomachines consisting of inner and outer membrane rings, known as a basal body, and an apparatus that enables bacteria to inject diverse effector proteins directly into the host cell cytoplasm, which participate in the regulation of host cell functions to benefit the bacterial survival and multiplication [3,4]. In the plant pathogenic bacteria, the T3SSs have attracted much attention due to their ability to elicit the hypersensitive response (HR), a plant defense mechanism characterized by rapid cell death, in resistant or non-host plants and induce disease symptoms in host plants [4,5]. In *Erwinia* *amylovora*, for instance, the T3SS is a major pathogenicity factor as the T3SS-deficient mutants are unable to cause fire blight disease in Rosaceous plant hosts [6,7]. Studies of the T3SS from *Pseudomonas* *syringae* pv. *tomato* DC3000, which causes bacterial speck of tomato and the model plant *Arabidopsis* *thaliana*, have provided valuable information for the understanding of host–microbe interactions by signifying the T3SS and its effectors as essential players involved in the repression of plant defense mechanisms during the bacterial infection [8,9,10].

As is the case for many T3SS-expressing pathogens, a growing number of studies have explored novel disease management strategies using the T3SS as a target for the inhibition of bacterial pathogenesis [11,12,13]. In the studies of plant pathogenic bacteria, small molecules that target and inhibit the T3SSs have been discovered. The soft rot plant pathogen *Dickeya* *dadantii* served as the model organism due to its well-characterized regulatory pathways of the T3SS. It has been well established that the T3SSs from plant pathogenic bacteria are encoded by the hypersensitive response and pathogenicity (*hrp*) operon [14]. Based on the similarities of operon organization and regulatory systems, plant pathogenic bacterial *hrp* gene clusters are divided into two groups [15,16]. The group I *hrp* gene cluster includes those of *P*. *syringae*, *E*. *amylovora*, and *Dickeya* spp., in which the alternative sigma factor HrpL is the master regulator that activates the expression of most *hrp* genes [17,18,19]. *Ralstonia* spp. and *Xanthomonas* spp. possess the group II *hrp* gene clusters, whose expression is heavily relied on an AraC-type transcriptional activator HrpX [20,21]. Genetic and molecular analyses have identified multiple important regulators that control the expression of T3SS in plant pathogenic bacteria, suggesting that the regulation of T3SS is a highly modulated process during the infection of plants by pathogens, and such regulation often occurs in a hierarchical manner.

In this review, we summarize our current understanding of the regulation of T3SS, focusing on bacterial second messengers, transcriptional regulators, and post-transcriptional regulators that control the T3SS in a model pathogen *D*. *dadantii*. Similar studies of T3SSs from other plant pathogens, such as *P*. *syringae* and *E*. *amylovora*, have been thoroughly reviewed elsewhere [22,23,24,25,26]. Furthermore, we discuss early innovations of the T3SS inhibitors and their modes of action in *D*. *dadantii* and *E*. *amylovora*, highlight the recent discovery of novel T3SS inhibitors in rice pathogen *X*. *oryzae* pv. *oryzae*, bacterial wilt pathogen *R*. *solanacearum*, and *P*. *syringae* pv. *tomato*, and provide remaining challenges and future perspectives on the application of T3SS inhibitors for managing plant diseases.

## 2. Regulation of T3SS in *D*. *dadantii*

**Figure 1:**
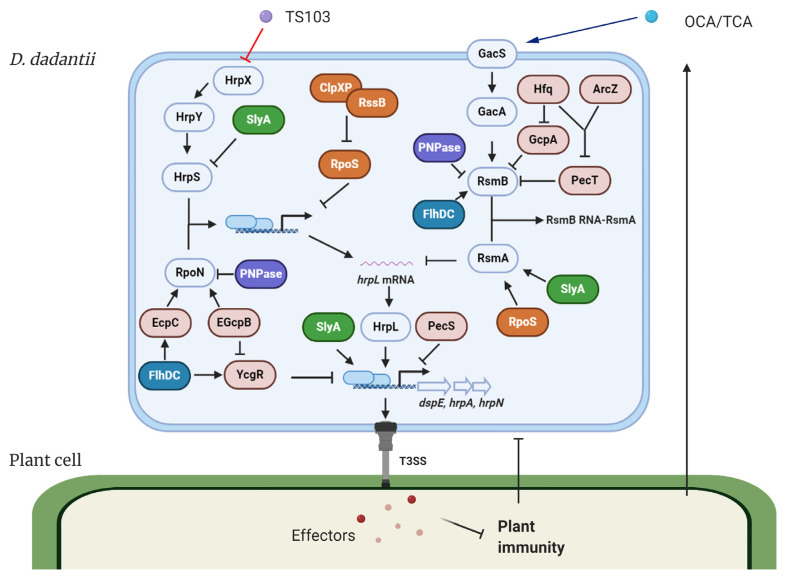
Model of the type III secretion system (T3SS) regulation and modes of action of T3SS inhibitors in *Dickeya* *dadantii*. The expression of T3SS master regulator HrpL is transcriptionally regulated by the HrpX/HrpY-HrpS-RpoN pathway and post-transcriptionally regulated by the GacS/GacA-RsmB-RsmA pathway. Several transcriptional regulators, including FlhDC, SlyA, PecS, PecT, and RpoS, regulate the expression of T3SS genes via targeting multiply key components in the T3SS regulatory pathways. Hfq and its dependent sRNA ArcZ form a feed-forward signaling cascade that positively control the expression of RsmB. PNPase degrades RsmB sRNA and is also required for the stability of *rpoN* mRNA. c-di-GMP signaling is involved in the FlhDC-mediated RpoN regulation and the Hfq-mediated RsmB regulation. T3SS injects bacterial effectors into the plant cells to suppress plant immune responses. On the other hand, plants secrete a variety of phenolic compounds that are able to regulate the T3SS in bacteria. For *D*. *dadantii*, two plant phenolic compounds, *o*-coumaric acid (OCA) and *t*-cinnamic acid (TCA), upregulate T3SS gene expression via the GacS/GacA-RsmB-RsmA-HrpL pathway. *Trans*-4-hydroxycinnamohdroxamic acid (TS103), a plant phenolic compound derivative, represses the expression of T3SS via the HrpX/HrpY-HrpS-RpoN-HrpL pathway. ⊥ represents negative control; → represents positive control.

The expression of T3SS in *D*. *dadantii* is governed by the master regulator HrpL. This alternative sigma factor activates T3SS gene expression by binding to the highly conserved *hrp* box (GGAACC-N_15/16_-CCACNNA) in their promoter regions. Genes such as *hrpA*, *hrpN*, and *dspE*, which encode the T3SS pilus protein, a harpin, and a virulence effector, respectively, are all transcribed in a HrpL-dependent manner [17,18,27,28]. The regulation of HrpL is achieved via two independent regulatory cascades in *D*. *dadantii* (Figure 1) [29,30,31,32]. Transcriptionally, HrpS is a σ^54^ (RpoN) enhancer binding protein, which binds the σ^54^ (RpoN)-containing RNA polymerase holoenzyme and initiates the expression of *hrpL*. A two-component signal transduction system (TCSTS) HrpX/HrpY is responsible for the activation of *hrpS* [29,33]. Post-transcriptionally, *hrpL* is regulated by RsmA, a small RNA-binding protein, which binds the 5′ untranslated region of *hrpL* mRNA and facilitates its degradation [34]. RsmB is a non-coding RNA containing multiple RsmA binding sites that bind RsmA proteins to antagonize their negative effect on *hrpL* mRNA, resulting in a positive effect on the downstream T3SS gene expression [33,35]. The GacS/GacA TCSTS has been shown to upregulate the production of RsmB RNA in *D*. *dadantii* [31].

### 2.1. Bacterial Second Messengers Regulate T3SS

*D*. *dadantii* can infect a wide range of host plants and has the ability to survive in soil and groundwater [36,37]. In the past decade, several regulators have been identified to be involved in the regulation of T3SS, implying a sophisticated regulatory network that allows *D*. *dadantii* to control its virulence gene expression to adapt to various environmental conditions. Bis-(3′-5′)-cyclic dimeric guanosine monophosphate (c-di-GMP), a ubiquitous bacterial second messenger in most major bacterial phyla, is one of the most critical and well-studied nodes of this network [38,39]. The metabolism of c-di-GMP is dependent on two kinds of enzymes: The GGDEF domain-containing diguanylate cyclase (DGC) enzymes that synthesize c-di-GMP from two molecules of guanosine-5′-triphosphate (GTP) and the EAL or HD-GYP domain-containing phosphodiesterase (PDE) enzymes that degrade c-di-GMP to 5′-phosphoguanylyl-(3′-5′)-guanosine or two molecules of guanosine monophosphate (GMP), respectively [40,41,42,43,44].

The presence of GGDEF and/or EAL domain protein-encoding genes is abundant in many Gram-negative bacteria. For example, *Escherichia* *coli* K-12 contains 29 genes, *Vibrio* *cholerae* contains 53, and *E*. *amylovora* contains 8 [45,46,47,48]. In *D*. *dadantii*, genomic analysis has identified twelve *gcp* (GGDEF domain-containing protein), four *ecp* (EAL domain-containing protein), and two *egcp* (EAL-GGDEF domain-containing protein) genes. Interestingly, genes encoding the HD-GYP domain-containing protein have not yet been annotated. Exploratory studies by Yi and colleagues demonstrated that two PDEs, EGcpB and EcpC, positively regulate the T3SS gene expression, swimming motility, and the production of pectate lyase (Pel), one of the major plant cell wall degrading enzymes [37,49], whereas negatively regulate biofilm formation, suggesting a pleiotropic effect of c-di-GMP in controlling *D*. *dadantii* pathogenesis [50,51]. These observations are also in agreement with reports from other bacteria, suggesting that bacterial second messenger c-di-GMP plays an essential role in promoting the transition from motile to sessile lifestyles [39,52]. Yi and colleagues further demonstrated that deletions of *egcpB* or *ecpC* cause elevated intracellular c-di-GMP levels, which in turn post-transcriptionally repress the expression of *rpoN* (Figure 1) [50]. Why *D*. *dadantii* utilizes two functionally redundant enzymes to modulate the same biological target remains unknown. To elucidate the function of DGCs in *D*. *dadantii*, Yuan and Tian et al. performed a phenotypical study on twelve *gcp* deletion mutants and found that a DGC GcpA synthesizes c-di-GMP to repress T3SS and Pel via negatively regulating *rsmB* at the post-transcriptional level and positively regulate RsmA protein levels [53]. H-NS, a nucleoid-associated protein known to activate Pel [54,55,56], is negatively regulated by GcpA at the level of post-transcription, which leads to the repression of *rsmB* [53]. Interestingly, H-NS is not involved in the regulation of T3SS by GcpA, possibly due to its role in modulating the DNA topology in *D*. *dadantii* [56]. Together, c-di-GMP has been shown to control the expression of T3SS in *D*. *dadantii* via two pathways: The RpoN-HrpL pathway and the RsmA/RsmB-HrpL pathway.

The expression of T3SS in plant pathogenic bacteria is known to be repressed in nutrient-rich media and induced in minimal media, which may correspond to the nutrient-deficient and low pH environment in plant apoplast [32,57,58]. To study the role of carbon source in T3SS regulation, the *D*. *dadantii* strain containing a transcriptional fusion of the *hrpN* gene to the *E. coli lacZ* gene was used as a reporter system [59]. The expression of the *hrpN*::*lacZ* fusion was under the control of the *hrpN* promoter and determined by the β-galactosidase assay. Nasser and colleagues reported that the expression of *hrpN*::*lacZ* fusion in *D*. *dadantii* was induced 5-fold in a minimal medium when supplemented with sucrose compared with glycerol [59], suggesting that the carbon source is involved in T3SS regulation. Yuan et al. recently showed that citrate, one of the major intermediates in the tricarboxylic acid (TCA) cycle, represses Pel production [60] and the T3SS gene expression (C.-H. Yang, unpublished data) in a c-di-GMP-dependent manner in *D*. *dadantii*. Deletions of TCA cycle enzymes reduce intracellular c-di-GMP levels in *D*. *dadantii* [60]; application of exogenous citrate induces intracellular c-di-GMP via promoting the expression of *gcpA* while repressing the expression of *egcpB* [60]. As the production of c-di-GMP has been reported to be induced by citrate in *P*. *fluorescens* [61], these data suggest that c-di-GMP might generally serve as an intracellular signal that regulates T3SSs in response to various environmental signals.

The nucleotide second messengers guanosine tetraphosphate (ppGpp) and guanosine pentaphosphate (pppGpp) are stringent response regulators that globally reprogram bacterial transcription via interacting with RNA polymerase and its binding protein DksA [62,63]. Several recent studies highlighted the role of (p)ppGpp in regulating T3SS in *E*. *amylovora* and *P*. *syringae*, showing that the (p)ppGpp-triggered T3SS gene expression might rely on the RpoN-HrpL cascade in *E*. *amylovora* [64,65,66,67]. However, whether this regulation occurs in *D*. *dadantii* has not yet been determined.

### 2.2. Transcriptional Regulators Control T3SS

The T3SS and flagellum are evolutionarily closely related due to their similarities in structure, function, and sequences of their main components [68,69,70,71]. In *P*. *syringae*, flagellin could be translocated into plant cells by the T3SS and induce immune responses [72]. Genetically, the master regulator of the flagellar assembly genes FlhDC has been shown to be essential for the activation of T3SS genes in *D*. *dadantii* and *Pectobacterium* *carotovorum* [51,73,74,75,76]. A detailed genetic study conducted by Yuan et al. demonstrated that *D*. *dadantii* FlhDC transcriptionally initiates the expression of *ecpC*, which regulates the T3SS through the c-di-GMP-mediated RpoN-HrpL pathway (Figure 1) [50,51]. Interestingly, the homolog of EcpC, named YhjH, in *E*. *coli* is also positively regulated by FlhDC. However, different from *E*. *coli*, the alternative sigma factor (σ^28^) FliA, which is under the control of FlhDC, is not required for the activation of *ecpC* via FlhDC in *D*. *dadantii* [51,77]. Additionally, since the consensus FlhDC binding sequence, AA(C/T)G(C/G)N_2-3_AAATA(A/G)CG [78,79], is not present in the promoter region of *ecpC*, the activation of *ecpC* by FlhDC might be indirect. FlhDC and YcgR, a PilZ domain protein, have also been shown to be involved in the regulation of T3SS through the RsmA/RsmB system (Figure 1) [51] and the function of YcgR has been shown to be activated by the binding to c-di-GMP [80,81]. However, in vitro studies demonstrated that their contributions to T3SS are not significant compared with the FlhDC-EcpC-RpoN-HrpL pathway [51].

The sigma factor RpoS is the master regulator for the stress response in bacteria [82]. In *E*. *coli*, RpoS is degraded by the ClpXP protease with the assistance of the recognition factor RssB [83,84,85]. RpoS from *P*. *carotovorum* has been reported to affect the production of Pel, T3SS gene *hrpN* expression, and virulence in plant through its regulation on RsmA [86]. Subsequent studies from *D*. *dadantii* and *E*. *amylovora* showed similar findings with a more detailed regulatory mechanism proposed in *D*. *dadantii*: The ClpXP-RssB-RpoS regulatory cascade controls the expression of *rsmA*, which post-transcriptionally affects HrpL; RpoS represses *hrpL* transcription without affecting HrpS or RpoN (Figure 1) [87,88], suggesting that a novel RpoS-HrpL pathway might exist in *D*. *dadantii*.

SlyA belongs to the member of the SlyA/MarR family transcriptional regulator [89]. In *D*. *dadantii*, SlyA was first characterized as a Pel regulator, and the deletion of *slyA* significantly reduced disease symptoms in planta [90]. Since SlyA has been reported to regulate the expression of T3SS in *Salmonella enterica serovar* Typhimurium [91], the role of SlyA homologue in controlling *D*. *dadantii* T3SS was investigated. Zou and colleagues reported that SlyA negatively regulates HrpL via two pathways: It upregulates the expression of *rsmA* and downregulates the transcription of *hrpS* that is independent of the TCSTS HrpX/HrpY (Figure 1). Interestingly, despite its negative impact on *hrpL*, SlyA positively regulates the expression of *hrp* regulon genes, such as *hrpA* and *hrpN*, in parallel with HrpL (Figure 1) [92], suggesting that multiple factors might be involved in the transcriptional regulation of T3SS genes in *D*. *dadantii*. Indeed, PecS, another MarR family transcriptional regulator [93,94], and PecT, a LysR family transcriptional regulator [37], have been shown to repress the transcription of *hrpN* (Figure 1) [59]. In vitro DNA-protein binding and DNase I footprinting analyses confirmed that PecS directly interacts with the promoter of *hrpN* to repress its transcription [59]. The mechanism of PecT-mediated regulation on *hrpN* remains unclear. Nevertheless, it will be interesting to know how and under what circumstances these transcriptional regulators modulate T3SS regulon gene expression in a cooperative or rather competitive manner.

### 2.3. Post-Transcriptional Regulators Control T3SS

Post-transcriptional regulation is an essential mechanism to control gene expression in bacteria [95,96]. Progress made in *D*. *dadantii* has identified several post-transcriptional regulators that control the expression of T3SS. Polynucleotide phosphorylase (PNPase) is one of the post-transcriptional regulators conserved in bacteria and eukaryotes [97,98,99]. Known as an exoribonuclease, PNPase is majorly involved in RNA decay [100]. The PNPase homologues in *Yersinia* spp. and *Salmonella* spp. have been reported to control T3SS [101,102]. The deletion of *pnpase* in *D*. *dadantii* significantly increased the transcriptional activities and mRNA levels of *hrpA*, *hrpN*, and *DspE*, suggesting that PNPase downregulates T3SS through HrpL in *D*. *dadantii* [103]. Further analyses confirmed that PNPase negatively regulates the stability of *rpoN* mRNA, which in turn affects the transcription of *hrpL*; PNPase also stimulates the decay of *hrpL* mRNA by reducing the amount of available RsmB transcripts (Figure 1) [103]. In *Salmonella* and *E*. *coli*, PNPase has been reported to be essential for the RNA decay of RsmB homologues CsrB [104,105].

The expression of RsmB was recently shown to be regulated by Hfq, an RNA chaperone [106,107], via a feed-forward signaling circuit in *D*. *dadantii* (Figure 1) [108]. Hfq and the Hfq-dependent small regulatory RNA (sRNA), ArcZ, repress the translation of *pecT*. PecT auto-inhibits its own transcription [109] and, more importantly, downregulates the transcription of *rsmB* that contributes to the Hfq-mediated regulation on the T3SS and Pel. As the PecT homologue HexA is also known to repress RsmB in *P*. *carotovorum* [110], it is reasonable to speculate that this regulation occurs at the level of transcription. RsmB is also post-transcriptionally regulated by Hfq since the deletion of *hfq* elevated the intracellular c-di-GMP levels owing to the increased productions of two DGCs, GcpA and GcpL. Both DGCs are required for the Hfq-mediated T3SS regulation. In *E*. *amylovora*, Zeng et al. found that Hfq and ArcZ were required for the virulence in host plants and the T3SS-dependent HR in non-host tobacco plants [111,112], but the mechanism has not yet been reported.

## 3. Discovery of T3SS Inhibitors in Plant Pathogens and Their Regulatory Mechanisms

Small molecules that could specifically inhibit the synthesis or functionality of the T3SS are referred to as T3SS inhibitors. Unlike traditional antibiotics that often target bacterial survival, T3SS inhibitors display negligible effects on bacterial growth, thus reducing the selective pressure for the development of resistance [12,113,114]. In plant pathogenic bacteria, extensive studies have led to the discovery of a group of plant-derived compounds and several chemically synthesized compounds that modulate the expression of T3SS in major plant pathogens (Table 1). Further studies detailed the mode of action of these compounds and investigated their potential for disease management.

### 3.1. Plant Phenolic Compounds as T3SS Inhibitors

Plant phenolic compounds are one of the most widespread secondary metabolites in plants. They range from low molecular weight and single aromatic ringed compounds to large and complex-polyphenols and are involved in diverse physiological activities in plants, such as pigmentation, growth, and defense mechanisms [123]. Plant phenolic compounds also function as signal molecules that either induce or repress the microbial gene expression during the interactions between plants and microbes [124]. As the expression of *D*. *dadantii* T3SS genes was induced in planta [125], Yang et al. discovered that two plant phenolic compounds, *o*-coumaric acid and *t*-cinnamic acid, positively regulated the expression of T3SS genes in *D*. *dadantii* [32]. *o*-coumaric acid upregulated *hrpL* at the post-transcriptional level via the RsmA/RsmB system and had no impact on the HrpX/HrpY-HrpS-HrpL pathway. Although *o*-coumaric acid and *t*-cinnamic acid are the biosynthetic precursors of salicylic acid, which plays an important role in plant defense responses [126,127], application of salicylic acid did not affect the expression of T3SS in *D*. *dadantii* [32].

To identify potential phenolic compounds as T3SS inhibitors, Li and colleagues evaluated the effects of 29 analogs and isomers of *o*-coumaric acid and *t*-cinnamic acid on the transcriptional activity of *hrpA*:*gfp* fusion in *D*. *dadantii*. One compound, *p*-coumaric acid, was shown to repress the expression of T3SS genes via the HrpS-HrpL pathway, as the addition of 100 µM *p*-coumaric acid significantly reduced the transcriptional activities and mRNA levels of *hrpS* and *hrpL* without affecting the bacterial growth [117]. *p*-coumaric acid is an intermediate in the phenylpropanoid biosynthesis pathway. Phenylpropanoids are plant secondary metabolites that act as defense molecules in response to microbial attack [128,129]. It is worth noting that the discovery of *p*-coumaric acid as a T3SS inhibitor has provided fundamental aspects for compound modification and greatly encouraged the innovation of chemically synthesized plant phenolic compound derivatives. A subsequent library screening of 50 *p*-coumaric acid derivatives identified *trans*-4-hydroxycinnamohydroxamic acid as a T3SS inhibitor in *D*. *dadantii*. The same study found that this synthetic compound increased the inhibitory potency towards T3SS by eightfold compared with the naturally occurring *p*-coumaric acid [118]. Furthermore, *trans*-4-hydroxycinnamohydroxamic acid was shown to repress *hrpL* transcriptionally through both the RpoN-HrpL pathway and the HrpX/HrpY-HrpS-HrpL pathway. *Trans*-4-hydroxycinnamohydroxamic acid repressed *rsmB* via unknown mechanisms, negatively contributing to the post-transcriptionally regulation of *hrpL* [118].

Using similar strategies as Li et al. [117,118], Khokhani and colleagues reported several plant phenolic compounds and derivatives that can modulate T3SS in *E*. *amylovora* [115]. *o*-coumaric acid and *t*-cinnamic acid, which induced the expression of T3SS in *D*. *dadantii* [32], repressed the *E*. *amylovora* T3SS. The T3SS inhibitors of *E*. *amylovora* also include benzoic acid, salicylic acid, and 4-methoxy-cinnamic acid. Benzoic acid negatively regulated *hrpS* expression, while 4-methoxy-cinnamic acid repressed HrpL, respectively. HR analysis demonstrated that co-infiltration of *E*. *amylovora* with benzoic acid or 4-methoxy-cinnamic acid reduced the HR development in *Nicotiana* *tabacum* cv. Xanthi leaves, suggesting that both phenolic compounds are functionally inhibiting T3SS *in* *planta*. Earlier studies on phenylpropanoid metabolism in *Vanilla* *planifolia* have reported that 4-methoxy-cinnamic acid is one of the intermediates in the biosynthetic conversion of cinnamic acid to benzoic acid [130]. Benzoic acid could be produced via the conversion of *t*-cinnamic acid to salicylic acid [131].

*X*. *oryzae* pv. *oryzae* is the causal agent of bacterial blight, one of the major rice diseases in the world. *X*. *oryzae* pv. *oryzicola*, which causes the bacterial leaf streak disease, is another rice pathogen [132]. Both pathogens process a T3SS encoded by the group II *hrp* gene clusters, in which HrpG and HrpX are two key regulators [16,21,30,133]. HrpG is a response regulator of the OmpR family of TCSTS, which positively regulates the expression of *hrpX*. HrpX is an AraC family transcriptional regulator responsible for activating the transcription of *hrp* genes [21,133]. By combining the transcriptional screening of the T3SS genes and HR assay in tobacco, Fan et al. identified four plant phenolic compounds, including *o*-coumaric acid, *trans*-2-methoxycinnamic acid, *trans*-2-phenylcyclopropane-1-carboxylic acid, and *trans*-2-methylcinnamic acid, actively inhibited the T3SS gene expression in vitro likely through the HrpG-HrpX regulatory cascade. Application of these T3SS inhibitors reduced the HR of *X*. *oryzae* pv. *oryzae* in non-host plants and the water soaking and disease symptoms of the pathogen in rice [116]. Since no impact on other virulence factors of *X*. *oryzae* pv. *oryzae*, such as the type II secretion system (T2SS), exopolysaccharide (EPS), and lipopolysaccharide (LPS) [134,135], was observed, the reduced virulence caused by the plant phenolic compounds in *X*. *oryzae* pv. *oryzae* is T3SS-dependent. A recent study was conducted to further understand the response of *X*. *oryzae* pv. *oryzae* to *o*-coumaric acid using transcriptomic analysis [136].

In *R*. *solanacearum*, the bacterial wilt pathogen of tomato, much progress has been made in identifying coumarins as T3SS inhibitors. Coumarins, consisting of fused benzene and α-pyrone rings, are a family of plant-derived secondary metabolites containing a large class of phenolic substances [137]. Members of the coumarins have been extensively studied mainly for their antimicrobial properties [138]. Umbelliferone is a 7-hydroxycoumarin that has recently been reported to repress the expression of *hrpG* and multiple T3SS regulon genes in *R*. *solanacearum* [119,120]. It reduced biofilm formation and suppressed the wilting disease process by reducing the colonization and proliferation of *R*. *solanacearum* *in* *planta* [120]. Besides, six plant phenolic compounds and derivatives, including *p*-coumaric acid, that repress the T3SS in *D*. *dadantii*, failed to modulate the expression of *R*. *solanacearum* T3SS genes [122].

### 3.2. Salicylidene Acylhydrazides and Their Derivatives as T3SS Inhibitors

Salicylidene acylhydrazides are one of the earliest T3SS inhibitors that were first identified through large-scale chemical screening approaches in *Yersinia* spp. [139,140,141]. Later studies have shown that salicylidene acylhydrazide family compounds actively against T3SSs from other human pathogenic bacteria, including *S*. *enterica*, *Shigella*, *Chlamydia*, *P*. *aeruginosa*, and enteropathogenic *E*. *coli* [142,143,144,145,146,147,148,149,150]. However, whether salicylidene acylhydrazides inhibit the plant pathogenic bacterial T3SSs remained unknown. To address this question, Yang and colleagues screened a small library of compounds. They found that several salicylidene acylhydrazides repressed the expression of T3SS genes, including the master regulator encoding gene *hrpL*, in *E*. *amylovora* [121]. Microarray analysis of *E*. *amylovora* treated with compound 3, also known as ME0054 [benzoic acid N′-(2,3,4-trihydroxy-benzylidene)-hydrazide] [141], confirmed its inhibitory impact on T3SS, as the majority of the 38 known T3SS genes was downregulated [121]. In addition, compound 3 suppressed the production of EPS amylovoran, one of the major pathogenicity factors of *E*. *amylovora* [7,151], and reduced the disease development of *E*. *amylovora* on crab apple flowers [121].

Puigvert and colleagues recently applied a similar approach to the T3SS of *R*. *solanacearum* to discover T3SS inhibitors [122]. Three synthetic salicylidene acylhydrazide derivatives, ME0054, ME0055 [4-nitrobenzoic acid N′-(2,4-dihydroxy-benzylidene)-hydrazide], and ME0177 [2-nitro-benzoic acid N′-(3,5-dichloro-2-hydroxy-benzylidene)-hydrazide] [141,152], were shown to inhibit the expression of T3SS genes through the inhibition of the regulator encoding gene *hrpB* [122]. All three salicylidene acylhydrazides could suppress the multiplication of *R*. *solanacearum* in planta and protect tomato from bacterial speck caused by *P*. *syringae* pv. *tomato* [122]. Since Yang et al. reported that ME0054 and ME0055 are T3SS inhibitors in *E*. *amylovora* [121], these findings suggest that salicylidene acylhydrazides and their derivatives are effective against different plant pathogenic bacteria.

## 4. Conclusions and Perspectives

Bacterial plant diseases, such as bacterial wilt, fire blight, soft rot, citrus greening, and bacterial leaf blight of rice, cause significant economic losses ($100M) annually on a global scale. Current control options are limited and involve applying chemicals, copper, and antibiotics, and biological control agents like bacteriophage [113,153]. In the field of fire blight management, for instance, three antibiotics have been used, including streptomycin, oxytetracycline, and a newly registered antibiotic kasugamycin. They are proven to be the most effective method in controlling fire blight in apple and pear orchards in the United States [154,155]. However, the widespread of resistance to antibiotics has placed major constraints on antibiotic usage. An increasing number of studies has implied the preexistence of antibiotic-resistance genes in environmental microbiome, such as soil, ground water, phyllosphere, and animal gut, before introducing antibiotics [114]. In addition, the extensive application of antibiotics in agriculture could greatly endanger human health as antibiotic-resistance genes are able to transfer between bacterial species via horizontal gene transfer [156]. Thus, the discovery and development of alternative control methods for controlling bacterial plant diseases are urgently needed.

T3SS is an essential virulence factor in many gram-negative bacterial pathogens [1,2]. Plant pathogenic bacteria use T3SSs to translocate various effector proteins into host cells to manipulate plant signaling behaviors and repress host immune responses [4]. The innovation of novel molecules that specifically target and inhibit primary virulence factors, such as T3SS, without lethal selective pressure serves as a compelling control option compared with conventional antibiotics. However, unlike antibiotics, often with known modes of action, the mechanisms of newly discovered virulence inhibitors are not well established in bacteria. This could be due to the lack of fundamental knowledge for the regulation of virulence factors and will be facilitated by analyzing the landscape of bacterial responses to virulence inhibitors at the whole-genome transcription level via next-generation sequencing techniques [136].

In the model microorganism *D*. *dadantii*, several regulatory components, including transcriptional and post-transcriptional regulators, sRNAs, and bacterial second messengers, have been reported to regulate the T3SS (Figure 1). The majority of these regulators, like c-di-GMP, are genetically and functionally conserved between different bacterial species, implicating that small molecules proven to be effective in one bacterium can also be applied in other bacteria. Several exemplary studies have already proved this concept via studying the impact of plant phenolic compounds and salicylidene acylhydrazides on T3SSs in various animal and plant pathogens [118,121,122]. Both small peptides and molecules (natural or synthetic) have been extensively studied in human pathogens *P*. *aeruginosa* and *V*. *cholerae* due to their impact on the c-di-GMP signaling. These molecules exert their function via binding to c-di-GMP directly, affecting the enzymatic activity of DGCs that synthesize c-di-GMP, or mimicking c-di-GMP as a competitor in bacteria [157,158,159,160]. However, similar studies have not been reported in plant pathogenic bacteria, and the idea of using small molecules that target c-di-GMP for the management of plant diseases needs to be further evaluated. On the other hand, controlling bacterial diseases using T3SS inhibitors may raise a concern whether they will interfere with the T3SS in some non-pathogenic or host beneficial bacteria that is also important for host interactions [161]. Ecologically, T3SS has been shown to contribute to the interaction between bacteria and fungi in soil and related habitats [162]. Thus, besides the capacity for disease management, the impact of T3SS inhibitors on the host microbiomes and the host plants should be monitored and evaluated.

Another challenge for the T3SS or any other virulence inhibitors is a successful implementation in the field. Unlike laboratory conditions, most commercially grown plants face fluctuating environmental conditions, such as sunlight, precipitation, and temperature. Therefore, it is important to evaluate the stability and efficacy of T3SS inhibitors under various environmental conditions. For example, a recent study showed that oxytetracycline and kasugamycin could be degraded by sunlight and early evening application is suggested to maximize the efficacy in controlling fire blight in the field [163]. In 2014 and 2015, we conducted field assays using a phenolic T3SS inhibitor, *trans*-4-phenylcinnamic acid, against fire blight on apple trees. Our results showed that it reduced blossom blight with an efficacy similar to kasugamycin at a concentration of 5 mM (Yang and Sundin, unpublished data), suggesting that application of T3SS inhibitor is a promising alternative method for controlling fire blight in the field. Meanwhile, trial application and evaluation of T3SS inhibitors on other bacterial plant diseases are planned. With more new discoveries, these antibiotic alternatives are expected to make agriculture more prepared for the upcoming challenges from continuously evolving pathogens.

## Figures and Tables

**Table 1:** Compounds that regulate T3SS in some major plant pathogens.

Compound	Structure	Known Modes of Action on T3SS in Plant Pathogens	References
*o*-coumaric acid	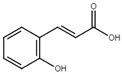	Induce T3SS via the RsmA/RsmB-HrpL pathway in *D*. *dadantii*; inhibit T3SS in *E*. *amylovora* and T3SS in *X*. *oryzae* pv. *oryzae* via the HrpG-HrpX regulatory cascade.	[32,115,116]
*t*-cinnamic acid	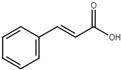	Induce T3SS in *D*. *dadantii*; inhibit T3SS in *E*. *amylovora*.	[32,115]
*p*-coumaric acid	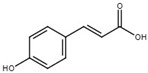	Inhibit T3SS via the HrpS-HrpL pathway in *D*. *dadantii*.	[117]
*trans*-4-hydroxycinnamodydroxamic acid	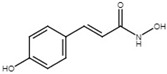	Inhibit T3SS via the RpoN-HrpL pathway, the HrpX/HrpY-HrpS-HrpL pathway, and the RsmB-HrpL pathway in *D*. *dadantii*.	[118]
Benzoic acid	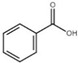	Inhibit T3SS via targeting HrpS in *E*. *amylovora*.	[115]
Salicylic acid	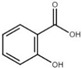	Inhibit T3SS in *E*. *amylovora*.	[115]
4-methoxy-cinnamic acid	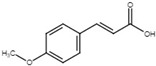	Inhibit T3SS via targeting HrpL in *E*. *amylovora*.	[115]
*trans*-2-methoxycinnamic acid	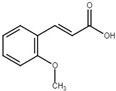	Inhibit T3SS via the HrpG-HrpX regulatory cascade in *X*. *oryzae* pv. *oryzae*.	[116]
*trans*-2-phenylcyclopropane-1-carboxylic-acid	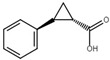	Inhibit T3SS via the HrpG-HrpX regulatory cascade in *X*. *oryzae* pv. *oryzae*.	[116]
*trans*-2-methylcinnamic acid	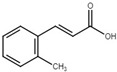	Inhibit T3SS via the HrpG-HrpX regulatory cascade in *X*. *oryzae* pv. *oryzae*.	[116]
Umbelliferone	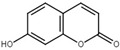	Inhibit T3SS via targeting HrpG in *R*. *solanacearum*.	[119,120]
Benzoic acid N′-(2,3,4-trihydroxy-benzylidene)-hydrazide	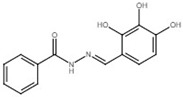	Inhibit multiple T3SS regulon genes in *E*. *amylovora*; inhibit T3SS via targeting HrpB in *R*. *solanacearum*.	[121,122]
4-nitrobenzoic acid N′-(2,4-dihydroxy-benzylidene)-hydrazide	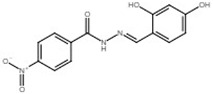	Inhibit T3SS via targeting HrpB in *R*. *solanacearum*; inhibit T3SS via targeting HrpL in *E*. *amylovora*.	[121,122]
2-nitro-benzoic acid N′-(3,5-dichloro-2-hydroxy-benzylidene)-hydrazide	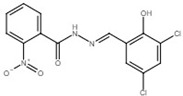	Inhibit T3SS via targeting HrpB in *R*. *solanacearum*.	[122]

## References

[1] Deng W., Marshall N.C., Rowland J.L., McCoy J.M., Worrall L.J., Santos A.S., Strynadka N.C., Finlay B.B. (2017). Assembly, structure, function and regulation of type III secretion systems. Nat. Rev. Microbiol..

[2] Hueck C.J. (1998). Type III protein secretion systems in bacterial pathogens of animals and plants. Microbiol. Mol. Biol. Rev..

[3] Coburn B., Sekirov I., Finlay B.B. (2007). Type III secretion systems and disease. Clin. Microbiol. Rev..

[4] Büttner D., He S.Y. (2009). Type III protein secretion in plant pathogenic bacteria. Plant. Physiol..

[5] Balint-Kurti P. (2019). The plant hypersensitive response: Concepts, control and consequences. Mol. Plant Pathol..

[6] Oh C.S., Kim J.F., Beer S.V. (2005). The Hrp pathogenicity island of *Erwinia*
*amylovora* and identification of three novel genes required for systemic infection. Mol. Plant. Pathol..

[7] Oh C.-S., Beer S.V. (2005). Molecular genetics of *Erwinia*
*amylovora* involved in the development of fire blight. FEMS Microbiol. Lett..

[8] Cunnac S., Lindeberg M., Collmer A. (2009). *Pseudomonas**syringae* type III secretion system effectors: Repertoires in search of functions. Curr. Opin. Microbiol..

[9] Jin L., Ham J.H., Hage R., Zhao W., Soto-Hernandez J., Lee S.Y., Paek S.-M., Kim M.G., Boone C., Coplin D.L. (2016). Direct and indirect targeting of PP2A by conserved bacterial type-III effector proteins. PLOS Pathog..

[10] Xin X.-F., Nomura K., Ding X., Chen X., Wang K., Aung K., Uribe F., Rosa B., Yao J., Chen J. (2015). *Pseudomonas**syringae* effector avirulence protein E localizes to the host plasma membrane and down-regulates the expression of the nonrace-specific disease resistance1/harpin-induced1-like13 gene required for antibacterial immunity in *Arabidopsis*. Plant. Physiol..

[11] Duncan M.C., Linington R.G., Auerbuch V. (2012). Chemical inhibitors of the type three secretion system: Disarming bacterial pathogens. Antimicrob. Agents Chemother..

[12] Pendergrass H.A., May A.E. (2019). Natural product type III secretion system inhibitors. Antibiotics.

[13] Gu L., Zhou S., Zhu L., Liang C., Chen X. (2015). Small-molecule inhibitors of the type III secretion system. Mol. Cells.

[14] Arnold D.L., Pitman A., Jackson R.W. (2003). Pathogenicity and other genomic islands in plant pathogenic bacteria. Mol. Plant Pathol..

[15] Troisfontaines P., Cornelis G.R. (2005). Type III secretion: More systems than you think. Physiology.

[16] Alfano J.R., Collmer A. (1997). The type III (Hrp) secretion pathway of plant pathogenic bacteria: Trafficking harpins, Avr proteins, and death. J. Bacteriol..

[17] Wei Z.-M., Beer S.V. (1995). *hrpL* activates *Erwinia amylovora hrp* gene transcription and is a member of the ECF subfamily of sigma factors. J. Bacteriol..

[18] Xiao Y., Hutcheson S.W. (1994). A single promoter sequence recognized by a newly identified alternate sigma factor directs expression of pathogenicity and host range determinants in *Pseudomonas*
*syringae*. J. Bacteriol..

[19] Xiao Y., Heu S., Yi J., Lu Y., Hutcheson S. (1994). Identification of a putative alternate sigma factor and characterization of a multicomponent regulatory cascade controlling the expression of *Pseudomonas*
*syringae* pv. *syringae* Pss61 *hrp* and *hrmA* genes. J. Bacteriol..

[20] Brito B., Aldon D., Barberis P., Boucher C., Genin S. (2002). A signal transfer system through three compartments transduces the plant cell contact-dependent signal controlling *Ralstonia*
*solanacearum*
*hrp* genes. Mol. Plant-Microbe Interact..

[21] Wengelnik K., Bonas U. (1996). HrpXv, an AraC-type regulator, activates expression of five of the six loci in the *hrp* cluster of *Xanthomonas*
*campestris* pv. vesicatoria. J. Bacteriol..

[22] McNally R.R., Zhao Y., Sundin G.W. (2015). Towards understanding fire blight: Virulence mechanisms and their regulation in *Erwinia*
*amylovora*. Bacteria-Plant Interactions: Advanced Research and Future Trends.

[23] Zhao Y., Sundin G. (2017). Exploring linear and cyclic (di)-nucleotides as messengers for regulation of T3SS and biofilm formation in *Erwinia*
*amylovora*. J. Plant. Pathol..

[24] Zhao Y. (2014). Genomics of *Erwinia*
*amylovora* and related *Erwinia* species associated with pome fruit trees. Genomics of Plant-Associated Bacteria.

[25] Malnoy M., Martens S., Norelli J.L., Barny M.-A., Sundin G.W., Smits T.H., Duffy B. (2012). Fire blight: Applied genomic insights of the pathogen and host. Annu. Rev. Phytopathol..

[26] Xie Y., Shao X., Deng X. (2019). Regulation of type III secretion system in *Pseudomonas*
*syringae*. Environ. Microbiol..

[27] Lam H.N., Chakravarthy S., Wei H.-L., BuiNguyen H., Stodghill P.V., Collmer A., Swingle B.M., Cartinhour S.W. (2014). Global analysis of the HrpL regulon in the plant pathogen *Pseudomonas*
*syringae* pv. *tomato* DC3000 reveals new regulon members with diverse functions. PLoS ONE.

[28] Yang S., Peng Q., Zhang Q., Zou L., Li Y., Robert C., Pritchard L., Liu H., Hovey R., Wang Q. (2010). Genome-Wide Identification of HrpL-Regulated Genes in the Necrotrophic Phytopathogen *Dickeya*
*dadantii* 3937. PLoS ONE.

[29] Yap M.-N., Yang C.-H., Barak J.D., Jahn C.E., Charkowski A.O. (2005). The *Erwinia*
*chrysanthemi* type III secretion system is required for multicellular behavior. J. Bacteriol..

[30] Tang X., Xiao Y., Zhou J.-M. (2006). Regulation of the type III secretion system in phytopathogenic bacteria. Mol. Plant.-Microbe Interact..

[31] Yang S., Peng Q., Zhang Q., Yi X., Choi C.J., Reedy R.M., Charkowski A.O., Yang C.-H. (2008). Dynamic regulation of GacA in type III secretion, pectinase gene expression, pellicle formation, and pathogenicity of *Dickeya*
*dadantii* (*Erwinia*
*chrysanthemi* 3937). Mol. Plant.-Microbe Interact..

[32] Yang S., Peng Q., San Francisco M., Wang Y., Zeng Q., Yang C.-H. (2008). Type III secretion system genes of *Dickeya*
*dadantii* 3937 are induced by plant phenolic acids. PLoS ONE.

[33] Chatterjee A., Cui Y., Chatterjee A.K. (2002). Regulation of *Erwinia*
*carotovora*
*hrpL_Ecc_* (sigma-*L_Ecc_*), Which Encodes an Extracytoplasmic Function Subfamily of Sigma Factor Required for Expression of the HRP Regulon. Mol. Plant.-Microbe Interact..

[34] Chatterjee A., Cui Y., Liu Y., Dumenyo C.K., Chatterjee A.K. (1995). Inactivation of *rsmA* leads to overproduction of extracellular pectinases, cellulases, and proteases in *Erwinia*
*carotovora* subsp. *carotovora* in the absence of the starvation/cell density-sensing signal, N-(3-oxohexanoyl)-L-homoserine lactone. Appl. Environ. Microbiol..

[35] Liu M.Y., Gui G., Wei B., Preston J.F., Oakford L., Yüksel Ü., Giedroc D.P., Romeo T. (1997). The RNA molecule CsrB binds to the global regulatory protein CsrA and antagonizes its activity in *Escherichia*
*coli*. J. Biol. Chem..

[36] Czajkowski R., Perombelon M.C., van Veen J.A., van der Wolf J.M. (2011). Control of blackleg and tuber soft rot of potato caused by *Pectobacterium* and *Dickeya* species: A review. Plant. Pathol..

[37] Reverchon S., Nasser W. (2013). *Dickeya* ecology, environment sensing and regulation of virulence programme. Environ. Microbiol. Rep..

[38] Römling U., Galperin M.Y., Gomelsky M. (2013). Cyclic di-GMP: The first 25 years of a universal bacterial second messenger. Microbiol. Mol. Biol. Rev..

[39] Hengge R. (2009). Principles of c-di-GMP signalling in bacteria. Nat. Rev. Microbiol..

[40] Paul R., Weiser S., Amiot N.C., Chan C., Schirmer T., Giese B., Jenal U. (2004). Cell cycle-dependent dynamic localization of a bacterial response regulator with a novel di-guanylate cyclase output domain. Genes Dev..

[41] Whiteley C.G., Lee D.-J. (2015). Bacterial diguanylate cyclases: Structure, function and mechanism in exopolysaccharide biofilm development. Biotechnol. Adv..

[42] Ryan R.P., Fouhy Y., Lucey J.F., Crossman L.C., Spiro S., He Y.-W., Zhang L.-H., Heeb S., Cámara M., Williams P. (2006). Cell-cell signaling in *Xanthomonas campestris* involves an HD-GYP domain protein that functions in cyclic di-GMP turnover. Proc. Natl. Acad. Sci. USA.

[43] Schmidt A.J., Ryjenkov D.A., Gomelsky M. (2005). The ubiquitous protein domain EAL is a cyclic diguanylate-specific phosphodiesterase: Enzymatically active and inactive EAL domains. J. Bacteriol..

[44] Tamayo R., Tischler A.D., Camilli A. (2005). The EAL domain protein VieA is a cyclic diguanylate phosphodiesterase. J. Biol. Chem..

[45] Povolotsky T.L., Hengge R. (2012). ‘Life-style’control networks in *Escherichia*
*coli*: Signaling by the second messenger c-di-GMP. J. Biotechnol..

[46] Waters C.M., Lu W., Rabinowitz J.D., Bassler B.L. (2008). Quorum sensing controls biofilm formation in *Vibrio*
*cholerae* through modulation of cyclic di-GMP levels and repression of *vpsT*. J. Bacteriol..

[47] Edmunds A.C., Castiblanco L.F., Sundin G.W., Waters C.M. (2013). Cyclic Di-GMP modulates the disease progression of *Erwinia*
*amylovora*. J. Bacteriol..

[48] Kharadi R.R., Castiblanco L.F., Waters C.M., Sundin G.W. (2019). Phosphodiesterase Genes Regulate Amylovoran Production, Biofilm Formation, and Virulence in *Erwinia*
*amylovora*. Appl. Environ. Microbiol..

[49] Hugouvieux-Cotte-Pattat N., Condemine G., Shevchik V.E. (2014). Bacterial pectate lyases, structural and functional diversity. Environ. Microbiol. Rep..

[50] Yi X., Yamazaki A., Biddle E., Zeng Q., Yang C.H. (2010). Genetic analysis of two phosphodiesterases reveals cyclic diguanylate regulation of virulence factors in *Dickeya*
*dadantii*. Mol. Microbiol..

[51] Yuan X., Khokhani D., Wu X., Yang F., Biener G., Koestler B.J., Raicu V., He C., Waters C.M., Sundin G.W. (2015). Cross-talk between a regulatory small RNA, cyclic-di-GMP signalling and flagellar regulator FlhDC for virulence and bacterial behaviours. Environ. Microbiol..

[52] Jenal U., Reinders A., Lori C. (2017). Cyclic di-GMP: Second messenger extraordinaire. Nat. Rev. Microbiol..

[53] Yuan X., Tian F., He C., Severin G.B., Waters C.M., Zeng Q., Liu F., Yang C.H. (2018). The diguanylate cyclase GcpA inhibits the production of pectate lyases via the H-NS protein and RsmB regulatory RNA in *Dickeya*
*dadantii*. Mol. Plant. Pathol..

[54] Nasser W., Faelen M., Hugouvieux-Cotte-Pattat N., Reverchon S. (2001). Role of the nucleoid-associated protein H-NS in the synthesis of virulence factors in the phytopathogenic bacterium *Erwinia chrysanthemi*. Mol. Plant.-Microbe Interact..

[55] Nasser W., Reverchon S. (2002). H-NS-dependent activation of pectate lyases synthesis in the phytopathogenic bacterium *Erwinia*
*chrysanthemi* is mediated by the PecT repressor. Mol. Microbiol..

[56] Ouafa Z.-A., Reverchon S., Lautier T., Muskhelishvili G., Nasser W. (2012). The nucleoid-associated proteins H-NS and FIS modulate the DNA supercoiling response of the pel genes, the major virulence factors in the plant pathogen bacterium *Dickeya*
*dadantii*. Nucleic Acids Res..

[57] Stauber J.L., Loginicheva E., Schechter L.M. (2012). Carbon source and cell density-dependent regulation of type III secretion system gene expression in *Pseudomonas*
*syringae* pathovar tomato DC3000. Res. Microbiol..

[58] Alfano J.R., Kim H.-S., Delaney T.P., Collmer A. (1997). Evidence that the *Pseudomonas*
*syringae* pv. *syringae*
*hrp*-linked *hrmA* gene encodes an Avr-like protein that acts in an *hrp*-dependent manner within tobacco cells. Mol. Plant-Microbe Interact..

[59] Nasser W., Reverchon S., Vedel R., Boccara M. (2005). PecS and PecT coregulate the synthesis of HrpN and pectate lyases, two virulence determinants in *Erwinia*
*chrysanthemi* 3937. Mol. Plant.-Microbe Interact..

[60] Yuan X., Zeng Q., Xu J., Severin G.B., Zhou X., Waters C.M., Sundin G.W., Ibekwe A.M., Liu F., Yang C.-H. (2020). Tricarboxylic Acid (TCA) Cycle Enzymes and Intermediates Modulate Intracellular Cyclic di-GMP Levels and the Production of Plant Cell Wall–Degrading Enzymes in Soft Rot Pathogen Dickeya dadantii. Mol. Plant-Microbe Interact. Mpmi.

[61] Giacalone D., Smith T.J., Collins A.J., Sondermann H., Koziol L.J., O’Toole G.A. (2018). Ligand-Mediated Biofilm Formation via Enhanced Physical Interaction between a Diguanylate Cyclase and Its Receptor. mBio.

[62] Ross W., Vrentas C.E., Sanchez-Vazquez P., Gaal T., Gourse R.L. (2013). The magic spot: A ppGpp binding site on *E*. *coli* RNA polymerase responsible for regulation of transcription initiation. Mol. Cell.

[63] Hauryliuk V., Atkinson G.C., Murakami K.S., Tenson T., Gerdes K. (2015). Recent functional insights into the role of (p)ppGpp in bacterial physiology. Nat. Rev. Microbiol..

[64] Yang H.-w., Yu M., Lee J.H., Chatnaparat T., Zhao Y. (2020). The stringent response regulator (p)ppGpp mediates virulence gene expression and survival in *Erwinia*
*amylovora*. BMC Genom..

[65] Ancona V., Lee J.H., Chatnaparat T., Oh J., Hong J.-I., Zhao Y. (2015). The bacterial alarmone (p)ppGpp activates the type III secretion system in *Erwinia*
*amylovora*. J. Bacteriol..

[66] Chatnaparat T., Li Z., Korban S.S., Zhao Y. (2015). The bacterial alarmone (p)ppGpp is required for virulence and controls cell size and survival of *Pseudomonas*
*syringae* on plants. Environ. Microbiol..

[67] Chatnaparat T., Li Z., Korban S.S., Zhao Y. (2015). The stringent response mediated by (p)ppGpp is required for virulence of *Pseudomonas*
*syringae* pv. *tomato* and its survival on tomato. Mol. Plant.-Microbe Interact..

[68] Young G.M., Schmiel D.H., Miller V.L. (1999). A new pathway for the secretion of virulence factors by bacteria: The flagellar export apparatus functions as a protein-secretion system. Proc. Natl. Acad. Sci. USA.

[69] Lee S.H., Galán J.E. (2004). *Salmonella* type III secretion-associated chaperones confer secretion-pathway specificity. Mol. Microbiol..

[70] Pallen M.J., Beatson S.A., Bailey C.M. (2005). Bioinformatics, genomics and evolution of non-flagellar type-III secretion systems: A Darwinian perpective. FEMS Microbiol. Rev..

[71] Erhardt M., Namba K., Hughes K.T. (2010). Bacterial nanomachines: The flagellum and type III injectisome. Cold Spring Harb. Perspect. Biol..

[72] Wei H.L., Chakravarthy S., Worley J.N., Collmer A. (2013). Consequences of flagellin export through the type III secretion system of *Pseudomonas*
*syringae* reveal a major difference in the innate immune systems of mammals and the model plant *Nicotiana*
*benthamiana*. Cell. Microbiol..

[73] Wang S., Fleming R.T., Westbrook E.M., Matsumura P., McKay D.B. (2006). Structure of the *Escherichia*
*coli* FlhDC complex, a prokaryotic heteromeric regulator of transcription. J. Mol. Biol..

[74] Aldridge P.D., Karlinsey J.E., Aldridge C., Birchall C., Thompson D., Yagasaki J., Hughes K.T. (2006). The flagellar-specific transcription factor, σ^28^, is the type III secretion chaperone for the flagellar-specific anti-σ^28^ factor FlgM. Genes Dev..

[75] Chilcott G.S., Hughes K.T. (2000). Coupling of Flagellar Gene Expression to Flagellar Assembly in *Salmonella*
*enterica* Serovar Typhimurium and *Escherichia*
*coli*. Microbiol. Mol. Biol. Rev..

[76] Cui Y., Chatterjee A., Yang H., Chatterjee A.K. (2008). Regulatory network controlling extracellular proteins in *Erwinia*
*carotovora* subsp. *carotovora*: FlhDC, the master regulator of flagellar genes, activates *rsmB* regulatory RNA production by affecting *gacA* and *hexA* (*lrhA*) expression. J. Bacteriol..

[77] Pesavento C., Becker G., Sommerfeldt N., Possling A., Tschowri N., Mehlis A., Hengge R. (2008). Inverse regulatory coordination of motility and curli-mediated adhesion in *Escherichia*
*coli*. Genes Dev..

[78] Claret L., Hughes C. (2002). Interaction of the atypical prokaryotic transcription activator FlhD_2_C_2_ with early promoters of the flagellar gene hierarchy. J. Mol. Biol..

[79] Stafford G.P., Ogi T., Hughes C. (2005). Binding and transcriptional activation of non-flagellar genes by the *Escherichia*
*coli* flagellar master regulator FlhD_2_C_2_. Microbiology.

[80] Fang X., Gomelsky M. (2010). A post-translational, c-di-GMP-dependent mechanism regulating flagellar motility. Mol. Microbiol..

[81] Paul K., Nieto V., Carlquist W.C., Blair D.F., Harshey R.M. (2010). The c-di-GMP binding protein YcgR controls flagellar motor direction and speed to affect chemotaxis by a “backstop brake” mechanism. Mol. Cell.

[82] Battesti A., Majdalani N., Gottesman S. (2011). The RpoS-mediated general stress response in *Escherichia*
*coli*. Annu. Rev. Microbiol..

[83] Lange R., Hengge-Aronis R. (1994). The cellular concentration of the sigma S subunit of RNA polymerase in *Escherichia*
*coli* is controlled at the levels of transcription, translation, and protein stability. Genes Dev..

[84] Zhou Y., Gottesman S., Hoskins J.R., Maurizi M.R., Wickner S. (2001). The RssB response regulator directly targets ςS for degradation by ClpXP. Genes Dev..

[85] Schweder T., Lee K.-H., Lomovskaya O., Matin A. (1996). Regulation of *Escherichia*
*coli* starvation sigma factor (sigma s) by ClpXP protease. J. Bacteriol..

[86] Mukherjee A., Cui Y., Ma W., Liu Y., Ishihama A., Eisenstark A., Chatterjee A.K. (1998). RpoS (sigma-S) controls expression of *rsmA*, a global regulator of secondary metabolites, harpin, and extracellular proteins in *Erwinia*
*carotovora*. J. Bacteriol..

[87] Li Y., Yamazaki A., Zou L., Biddle E., Zeng Q., Wang Y., Lin H., Wang Q., Yang C.-H. (2010). ClpXP protease regulates the type III secretion system of *Dickeya*
*dadantii* 3937 and is essential for the bacterial virulence. Mol. Plant.-Microbe Interact..

[88] Lee J.H., Zhao Y. (2017). ClpXP-Dependent RpoS Degradation Enables Full Activation of Type III Secretion System, Amylovoran Production, and Motility in *Erwinia*
*amylovora*. Phytopathology.

[89] Ellison D.W., Miller V.L. (2006). Regulation of virulence by members of the MarR/SlyA family. Curr. Opin. Microbiol..

[90] Haque M.M., Kabir M.S., Aini L.Q., Hirata H., Tsuyumu S. (2009). SlyA, a MarR family transcriptional regulator, is essential for virulence in *Dickeya*
*dadantii* 3937. J. Bacteriol..

[91] Linehan S.A., Rytkönen A., Yu X.-J., Liu M., Holden D.W. (2005). SlyA regulates function of *Salmonella* pathogenicity island 2 (SPI-2) and expression of SPI-2-associated genes. Infect. Immun..

[92] Zou L., Zeng Q., Lin H., Gyaneshwar P., Chen G., Yang C.-H. (2012). SlyA regulates type III secretion system (T3SS) genes in parallel with the T3SS master regulator HrpL in *Dickeya*
*dadantii* 3937. Appl. Environ. Microbiol..

[93] Hommais F., Oger-Desfeux C., Van Gijsegem F., Castang S., Ligori S., Expert D., Nasser W., Reverchon S. (2008). PecS is a global regulator of the symptomatic phase in the phytopathogenic bacterium *Erwinia*
*chrysanthemi* 3937. J. Bacteriol..

[94] Reverchon S., Rouanet C., Expert D., Nasser W. (2002). Characterization of indigoidine biosynthetic genes in *Erwinia*
*chrysanthemi* and role of this blue pigment in pathogenicity. J. Bacteriol..

[95] Romeo T., Vakulskas C.A., Babitzke P. (2013). Post-transcriptional regulation on a global scale: Form and function of Csr/Rsm systems. Environ. Microbiol..

[96] Van Assche E., Van Puyvelde S., Vanderleyden J., Steenackers H.P. (2015). RNA-binding proteins involved in post-transcriptional regulation in bacteria. Front. Microbiol..

[97] Kudla J., Hayes R., Gruissem W. (1996). Polyadenylation accelerates degradation of chloroplast mRNA. Embo J..

[98] Leszczyniecka M., Kang D.-c., Sarkar D., Su Z.-z., Holmes M., Valerie K., Fisher P.B. (2002). Identification and cloning of human polynucleotide phosphorylase, *hPNPase^old-35^*, in the context of terminal differentiation and cellular senescence. Proc. Natl. Acad. Sci. USA.

[99] Kinscherf T., Apirion D. (1975). Polynucleotide phosphorylase can participate in decay of mRNA in *Escherichia*
*coli* in the absence of ribonuclease II. Mol. Gen. Genet. MGG..

[100] Cameron T.A., Matz L.M., De Lay N.R. (2018). Polynucleotide phosphorylase: Not merely an RNase but a pivotal post-transcriptional regulator. PLoS Genet..

[101] Rosenzweig J.A., Chromy B., Echeverry A., Yang J., Adkins B., Plano G.V., McCutchen-Maloney S., Schesser K. (2007). Polynucleotide phosphorylase independently controls virulence factor expression levels and export in *Yersinia* spp.. FEMS Microbiol. Lett..

[102] Ygberg S.E., Clements M.O., Rytkönen A., Thompson A., Holden D.W., Hinton J.C., Rhen M. (2006). Polynucleotide phosphorylase negatively controls spv virulence gene expression in *Salmonella enterica*. Infect. Immun..

[103] Zeng Q., Ibekwe A.M., Biddle E., Yang C.-H. (2010). Regulatory mechanisms of exoribonuclease PNPase and regulatory small RNA on T3SS of *Dickeya*
*dadantii*. Mol. Plant.-Microbe Interact..

[104] Viegas S.C., Pfeiffer V., Sittka A., Silva I.s.J., Vogel J., Arraiano C.M. (2007). Characterization of the role of ribonucleases in *Salmonella* small RNA decay. Nucleic Acids Res..

[105] Dressaire C., Pobre V., Laguerre S., Girbal L., Arraiano C.M., Cocaign-Bousquet M. (2018). PNPase is involved in the coordination of mRNA degradation and expression in stationary phase cells of *Escherichia*
*coli*. BMC Genom..

[106] Chao Y., Vogel J. (2010). The role of Hfq in bacterial pathogens. Curr. Opin. Microbiol..

[107] Vogel J., Luisi B.F. (2011). Hfq and its constellation of RNA. Nat. Rev. Microbiol..

[108] Yuan X., Zeng Q., Khokhani D., Tian F., Severin G.B., Waters C.M., Xu J., Zhou X., Sundin G.W., Ibekwe A.M. (2019). A Feed-forward signaling circuit controls bacterial virulence through linking cyclic di-GMP and two mechanistically distinct sRNAs; ArcZ and RsmB. Environ. Microbiol..

[109] Castillo A., Reverchon S. (1997). Characterization of the *pecT* control region from *Erwinia chrysanthemi* 3937. J. Bacteriol..

[110] Mukherjee A., Cui Y., Ma W., Liu Y., Chatterjee A.K. (2000). *hexA* of *Erwinia*
*carotovora* ssp. *carotovora* strain Ecc71 negatively regulates production of RpoS and *rsmB* RNA, a global regulator of extracellular proteins, plant virulence and the quorum-sensing signal, *N*-(3-oxohexanoyl)-L-homoserine lactone. Environ. Microbiol..

[111] Zeng Q., Sundin G.W. (2014). Genome-wide identification of Hfq-regulated small RNAs in the fire blight pathogen *Erwinia*
*amylovora* discovered small RNAs with virulence regulatory function. BMC Genom..

[112] Zeng Q., McNally R.R., Sundin G.W. (2013). Global small RNA chaperone Hfq and regulatory small RNAs are important virulence regulators in *Erwinia*
*amylovora*. J. Bacteriol..

[113] Sundin G.W., Castiblanco L.F., Yuan X., Zeng Q., Yang C.H. (2016). Bacterial disease management: Challenges, experience, innovation and future prospects: Challenges in bacterial molecular plant pathology. Mol. Plant. Pathol..

[114] Sundin G.W., Wang N. (2018). Antibiotic Resistance in Plant-Pathogenic Bacteria. Annu. Rev. Phytopathol..

[115] Khokhani D., Zhang C., Li Y., Wang Q., Zeng Q., Yamazaki A., Hutchins W., Zhou S.-S., Chen X., Yang C.-H. (2013). Discovery of plant phenolic compounds that act as type III secretion system inhibitors or inducers of the fire blight pathogen, *Erwinia*
*amylovora*. Appl. Environ. Microbiol..

[116] Fan S., Tian F., Li J., Hutchins W., Chen H., Yang F., Yuan X., Cui Z., Yang C.H., He C. (2017). Identification of phenolic compounds that suppress the virulence of *Xanthomonas*
*oryzae* on rice via the type III secretion system. Mol. Plant Pathol..

[117] Li Y., Peng Q., Selimi D., Wang Q., Charkowski A.O., Chen X., Yang C.-H. (2009). The plant phenolic compound p-coumaric acid represses gene expression in the *Dickeya*
*dadantii* type III secretion system. Appl. Environ. Microbiol..

[118] Li Y., Hutchins W., Wu X., Liang C., Zhang C., Yuan X., Khokhani D., Chen X., Che Y., Wang Q. (2015). Derivative of plant phenolic compound inhibits the type III secretion system of *Dickeya*
*dadantii* via HrpX/HrpY two-component signal transduction and Rsm systems. Mol. Plant. Pathol..

[119] Yang L., Ding W., Xu Y., Wu D., Li S., Chen J., Guo B. (2016). New insights into the antibacterial activity of hydroxycoumarins against *Ralstonia*
*solanacearum*. Mol. Cells.

[120] Yang L., Li S., Qin X., Jiang G., Chen J., Li B., Yao X., Liang P., Zhang Y., Ding W. (2017). Exposure to umbelliferone reduces *Ralstonia*
*solanacearum* biofilm formation, transcription of type III secretion system regulators and effectors and virulence on tobacco. Front. Microbiol..

[121] Yang F., Korban S.S., Pusey P.L., Elofsson M., Sundin G.W., Zhao Y. (2014). Small-molecule inhibitors suppress the expression of both type III secretion and amylovoran biosynthesis genes in *Erwinia*
*amylovora*. Mol. Plant Pathol..

[122] Puigvert M., Solé M., López-Garcia B., Coll N.S., Beattie K.D., Davis R.A., Elofsson M., Valls M. (2019). Type III secretion inhibitors for the management of bacterial plant diseases. Mol. Plant Pathol..

[123] Velderrain-Rodríguez G., Palafox-Carlos H., Wall-Medrano A., Ayala-Zavala J., Chen C.O., Robles-Sánchez M., Astiazaran-García H., Alvarez-Parrilla E., González-Aguilar G. (2014). Phenolic compounds: Their journey after intake. Food Funct..

[124] Siqueira J.O., Nair M.G., Hammerschmidt R., Safir G.R., Putnam A.R. (1991). Significance of phenolic compounds in plant-soil-microbial systems. Crit. Rev. Plant. Sci..

[125] Yang S., Perna N.T., Cooksey D.A., Okinaka Y., Lindow S.E., Ibekwe A.M., Keen N.T., Yang C.-H. (2004). Genome-wide identification of plant-upregulated genes of *Erwinia*
*chrysanthemi* 3937 using a GFP-based IVET leaf array. Mol. Plant-Microbe Interact..

[126] Montesano M., Brader G., Ponce de León I., Palva E.T. (2005). Multiple defence signals induced by *Erwinia*
*carotovora* ssp. *carotovora* elicitors in potato. Mol. Plant Pathol..

[127] Vidal S., de León I.P., Denecke J., Palva E.T. (1997). Salicylic acid and the plant pathogen *Erwinia*
*carotovora* induce defense genes via antagonistic pathways. Plant. J..

[128] Dixon R.A., Paiva N.L. (1995). Stress-induced phenylpropanoid metabolism. Plant Cell.

[129] Feys B.J., Parker J.E. (2000). Interplay of signaling pathways in plant disease resistance. Trends Genet..

[130] Funk C., Brodelius P.E. (1990). Phenylpropanoid metabolism in suspension cultures of *Vanilla*
*planifolia* Andr.: III. Conversion of 4-methoxycinnamic acids into 4-hydroxybenzoic acids. Plant. Physiol..

[131] Yalpani N., Raskin I. (1993). Salicylic acid: A systemic signal in induced plant disease resistance. Trends Microbiol..

[132] Niño-Liu D.O., Ronald P.C., Bogdanove A.J. (2006). *Xanthomonas oryzae* pathovars: Model pathogens of a model crop. Mol. Plant Pathol..

[133] Wengelnik K., Van den Ackerveken G., Bonas U. (1996). HrpG, a key hrp regulatory protein of *Xanthomonas*
*campestris* pv. *vesicatoria* ls homologous to two-component response regulators. Mol. Plant-Microbe Interact..

[134] Das A., Rangaraj N., Sonti R.V. (2009). Multiple adhesin-like functions of *Xanthomonas*
*oryzae* pv. *oryzae* are involved in promoting leaf attachment, entry, and virulence on rice. Mol. Plant-Microbe Interact..

[135] Büttner D., Bonas U. (2010). Regulation and secretion of *Xanthomonas* virulence factors. Fems Microbiol. Rev..

[136] Fan S., Tian F., Fang L., Yang C.-H., He C. (2019). Transcriptional responses of *Xanthomonas*
*oryzae* pv. *oryzae* to type III secretion system inhibitor ortho-coumaric acid. BMC Microbiol..

[137] Stringlis I.A., De Jonge R., Pieterse C.M. (2019). The age of coumarins in plant–microbe interactions. Plant. Cell Physiol..

[138] Venugopala K.N., Rashmi V., Odhav B. (2013). Review on natural coumarin lead compounds for their pharmacological activity. BioMed Res. Int..

[139] Kauppi A.M., Nordfelth R., Uvell H., Wolf-Watz H., Elofsson M. (2003). Targeting bacterial virulence: Inhibitors of type III secretion in *Yersinia*. Chem. Biol..

[140] Kauppi A.M., Nordfelth R., Hägglund U., Wolf-Watz H., Elofsson M. (2004). Salicylanilides are potent inhibitors of type III secretion in *Yersinia*. The Genus Yersinia.

[141] Nordfelth R., Kauppi A.M., Norberg H., Wolf-Watz H., Elofsson M. (2005). Small-molecule inhibitors specifically targeting type III secretion. Infect. Immun..

[142] Muschiol S., Bailey L., Gylfe Å., Sundin C., Hultenby K., Bergström S., Elofsson M., Wolf-Watz H., Normark S., Henriques-Normark B. (2006). A small-molecule inhibitor of type III secretion inhibits different stages of the infectious cycle of *Chlamydia*
*trachomatis*. Proc. Natl. Acad. Sci. USA.

[143] Muschiol S., Normark S., Henriques-Normark B., Subtil A. (2009). Small molecule inhibitors of the *Yersinia* type III secretion system impair the development of *Chlamydia* after entry into host cells. BMC Microbiol..

[144] Veenendaal A.K., Sundin C., Blocker A.J. (2009). Small-molecule type III secretion system inhibitors block assembly of the *Shigella* type III secreton. J. Bacteriol..

[145] Wolf K., Betts H., Chellas-Gery B., Hower S., Linton C., Fields K. (2006). Treatment of *Chlamydia trachomatis* with a small molecule inhibitor of the *Yersinia* type III secretion system disrupts progression of the chlamydial developmental cycle. Mol. Microbiol..

[146] Bailey L., Gylfe Å., Sundin C., Muschiol S., Elofsson M., Nordström P., Henriques-Normark B., Lugert R., Waldenström A., Wolf-Watz H. (2007). Small molecule inhibitors of type III secretion in *Yersinia* block the *Chlamydia*
*pneumoniae* infection cycle. FEBS Lett..

[147] Tree J.J., Wang D., McInally C., Mahajan A., Layton A., Houghton I., Elofsson M., Stevens M.P., Gally D.L., Roe A.J. (2009). Characterization of the effects of salicylidene acylhydrazide compounds on type III secretion in *Escherichia*
*coli* O157: H7. Infect. Immun..

[148] Hudson D.L., Layton A.N., Field T.R., Bowen A.J., Wolf-Watz H., Elofsson M., Stevens M.P., Galyov E.E. (2007). Inhibition of type III secretion in *Salmonella*
*enterica* serovar Typhimurium by small-molecule inhibitors. Antimicrob. Agents Chemother..

[149] Negrea A., Bjur E., Ygberg S.E., Elofsson M., Wolf-Watz H., Rhen M. (2007). Salicylidene acylhydrazides that affect type III protein secretion in *Salmonella*
*enterica* serovar typhimurium. Antimicrob. Agents Chemother..

[150] Anantharajah A., Buyck J.M., Sundin C., Tulkens P.M., Mingeot-Leclercq M.-P., Van Bambeke F. (2017). Salicylidene acylhydrazides and hydroxyquinolines act as inhibitors of type three secretion systems in *Pseudomonas*
*aeruginosa* by distinct mechanisms. Antimicrob. Agents Chemother..

[151] Bellemann P., Geider K. (1992). Localization of transposon insertions in pathogenicity mutants of *Erwinia*
*amylovora* and their biochemical characterization. Microbiology.

[152] Dahlgren M.K., Zetterström C.E., Gylfe Å., Linusson A., Elofsson M. (2010). Statistical molecular design of a focused salicylidene acylhydrazide library and multivariate QSAR of inhibition of type III secretion in the Gram-negative bacterium *Yersinia*. Bioorganic Med. Chem..

[153] Buttimer C., McAuliffe O., Ross R.P., Hill C., O’Mahony J., Coffey A. (2017). Bacteriophages and bacterial plant diseases. Front. Microbiol..

[154] Stockwell V., Duffy B. (2012). Use of antibiotics in plant agriculture. Rev. Sci. Et Tech. Off. Int. Des. Epizoot..

[155] McGhee G.C., Sundin G.W. (2011). Evaluation of kasugamycin for fire blight management, effect on nontarget bacteria, and assessment of kasugamycin resistance potential in *Erwinia*
*amylovora*. Phytopathology.

[156] Benveniste R., Davies J. (1973). Aminoglycoside antibiotic-inactivating enzymes in actinomycetes similar to those present in clinical isolates of antibiotic-resistant bacteria. Proc. Natl. Acad. Sci. USA.

[157] Sambanthamoorthy K., Sloup R.E., Parashar V., Smith J.M., Kim E.E., Semmelhack M.F., Neiditch M.B., Waters C.M. (2012). Identification of small molecules that antagonize diguanylate cyclase enzymes to inhibit biofilm formation. Antimicrob. Agents Chemother..

[158] Kim B., Park J.-S., Choi H.-Y., Yoon S.S., Kim W.-G. (2018). Terrein is an inhibitor of quorum sensing and c-di-GMP in *Pseudomonas*
*aeruginosa*: A connection between quorum sensing and c-di-GMP. Sci. Rep..

[159] Hee C.-S., Habazettl J., Schmutz C., Schirmer T., Jenal U., Grzesiek S. (2020). Intercepting second-messenger signaling by rationally designed peptides sequestering c-di-GMP. Proc. Natl. Acad. Sci. USA.

[160] Cho K.H., Tryon R.G., Kim J.-H. (2020). Screening for Diguanylate Cyclase (DGC) Inhibitors Mitigating Bacterial Biofilm Formation. Front. Chem..

[161] Zamioudis C., Pieterse C.M. (2012). Modulation of host immunity by beneficial microbes. Mol. Plant.-Microbe Interact..

[162] Nazir R., Mazurier S., Yang P., Lemanceau P., Van Elsas J.D. (2017). The ecological role of type three secretion systems in the interaction of bacteria with fungi in soil and related habitats is diverse and context-dependent. Front. Microbiol..

[163] Slack S., Walters K.J., Outwater C., Sundin G.W. (2020). Effect of kasugamycin, oxytetracycline, and streptomycin on in-orchard population dynamics of *Erwinia*
*amylovora* on apple flower stigmas. Plant. Dis..

